# Brief psychological interventions for schizophrenia: a systematic review and meta-analysis

**DOI:** 10.1017/S0033291725001126

**Published:** 2025-05-13

**Authors:** Blue Pike, Leire Ambrosio, Lyn Ellett

**Affiliations:** 1 Recovery Service, Hampshire and Isle of Wight Healthcare NHS Foundation Trust, Isle of Wight, UK; 2 School of Health and Life Sciences, University of Southampton, Southampton, UK; 3 School of Psychology, University of Southampton, Southampton, UK

**Keywords:** brief intervention, CBT, meta-analysis, schizophrenia, systematic review

## Abstract

**Background:**

Although cognitive behavioral therapy for people diagnosed with schizophrenia (CBTp) is recommended in clinical guidelines internationally, rates of implementation are low. One consequence of this has been the development of brief individual psychological interventions, which are shorter than the recommended minimum of 16 sessions for CBTp. This article is the first to systematically identify the brief interventions that exist for people diagnosed with schizophrenia and to determine their effectiveness using meta-analysis.

**Methods:**

Five electronic databases (PsycINFO, MEDLINE, CINAHL, EMBASE, and Web of Science) were searched for peer-reviewed randomized controlled trials or experimental studies of brief individual psychological interventions delivered in community settings. Random effects meta-analysis was used to integrate effect sizes, due to the heterogeneity of included studies.

**Results:**

Fourteen studies were identified (n = 1,382) that measured thirty clinical outcomes and included six intervention types - brief CBT, memory training, digital motivation support, reasoning training, psychoeducation, and virtual reality. Collectively, brief psychological interventions were found to be effective for psychotic symptoms (SMD −0.285, p < 0.01), paranoia (SMD −0.277, p < 0.05), data gathering (SMD 0.38, p < 0.01), depression (SMD −0.906, p < 0.05) and wellbeing (SMD 0.405, p < 0.01). For intervention types, brief CBT was effective for psychotic symptoms (SMD −0.32, p < .001), and reasoning training was effective for data gathering (SMD 0.38, p < 0.01).

**Conclusions:**

Overall, the evidence suggests that brief psychological interventions are effective for several key difficulties associated with schizophrenia, providing an opportunity to improve both access to, and choice of, treatment for individuals diagnosed with schizophrenia.

## Introduction

Worldwide, access to effective treatment for schizophrenia is extremely limited, with only 31.3% receiving specialist care for psychosis (WHO, 2022). Cognitive Behavior Therapy for psychosis (CBTp) is one of the first-line treatments for schizophrenia recommended internationally in clinical guidelines, for example, in England and Wales, Germany, Canada, Australia, and New Zealand (Australian, 2005; Dixon et al., 2010; Gaebel, Riesbeck, & Wobrock, 2011; NICE, 2014; Norman, Lecomte, Addington, & Anderson, 2017). In the UK, NICE (2014) recommends a minimum of 16 sessions of CBTp (NICE., 2014). A recent review in the Netherlands found implementation rates for CBTp of 19–24% with a third of these treatments provided by psychologists who were not trained in CBTp (van de Ven et al., 2025). However, rates of implementation for CBTp are still below recommended levels with wide variation in rates found (Ince, Haddock, & Tai, 2016), with a review of international implementation rates for CBTp finding a pooled prevalence rate of 24% (Burgess-Barr et al., 2023).

There are a number of factors that contribute to poor access to CBTp, including constraints in service delivery due to increasing demand (Cantor, 2022) and a scarcity of trained therapists (O’Connor et al., 2018). Beyond the UK, other factors cited include a lack of appropriate health insurance (Chamberlin, 2004) and limited access to health facilities (Dye, Reeder., & Terry, 2013). One consequence of this has been the development of more targeted treatments that require fewer sessions, referred to as ‘brief interventions’ (Sijbrandij, Kleiboer, & Farooq, 2020). Brief CBT interventions have been shown to be effective for anxiety and depression (Wakefield et al., 2021) and are recommended by the National Institute for Health and Care Excellence (NICE, 2022), and there is emerging evidence that brief CBT interventions are also effective for people with psychosis (Hazell, Hayward, Cavanagh, & Strauss, 2016). Additionally, some studies have found that using more discrete components of CBT has been effective for treating individual psychotic symptoms, including persecutory delusions (Freeman et al., 2016), and auditory hallucinations (Craig et al., 2018).

A systematic review and meta-analysis of low intensity CBTp of 10 controlled trials with <16 sessions found a small-medium effect (*d* = −0.46), which was maintained at follow-up (*d* = −0.40) for symptoms of psychosis (Hazell et al., 2016). The effect sizes found for brief CBTp were similar to those found in meta-analyses of standard CBTp, suggesting that brief interventions are beneficial for people diagnosed with schizophrenia (Hazell et al., 2016). However, this review was limited in its focus to brief CBT interventions only, and there is no current review summarizing the full range of brief interventions available for people diagnosed with schizophrenia. Therefore, the aim of this study was to review the range and effectiveness of brief (<16 sessions) interventions for schizophrenia. The review focuses on community interventions as factors such as acuity and length of admission can impact the type and duration of interventions that are accessible for inpatients (Duncan et al., 2021; Johnson et al., 2022). The review addressed the following research questions: 1. What brief individual psychological interventions exist for people diagnosed with schizophrenia? 2. How effective are brief psychological interventions for reducing psychotic symptoms? 3. How effective are brief psychological interventions for improving secondary outcomes?

## Methods

### Protocol and search strategy

The protocol for this review was registered with PROSPERO on November 24, 2023 (PROSPERO registration CRD42023479319).

Five electronic databases were searched in December 2023, including PsycINFO, MEDLINE, CINAHL, EMBASE, and Web of Science. The search terms Schizophrenia* OR Schizophrenia (MeSH term) OR psychosis OR psychoses OR psychotic disorder OR schizophrenic disorder OR hallucination* OR voice* OR auditory hallucination* OR delusion* AND brief intervention* OR short-term were used in abstract and title, and limitations were added for papers that included adults 18+ years, were peer reviewed and published in English language. Search results were exported from each database to Rayyan (https://www.rayyan.ai/) where duplicate records were removed.

### Inclusion and exclusion criteria

Inclusion criteria: (1) randomized controlled trials (RCT) or randomized experimental studies, (2) studies that include populations of individuals with a schizophrenia spectrum diagnosis, (3) community treatment, and (4) individual interventions with a duration of less than 16 sessions.

Exclusion criteria: (1) study focus is medication (2) focus is on caregivers or staff, (3) qualitative study or case study, (4) focus is on drug, smoking or alcohol use, (5) focus is on inpatient care (including forensic setting) or transfer to community, (6) book chapters/editorials and existing reviews, (7) secondary analysis of already published datasets, and (8) group interventions.

### Study selection

**Figure 1.**
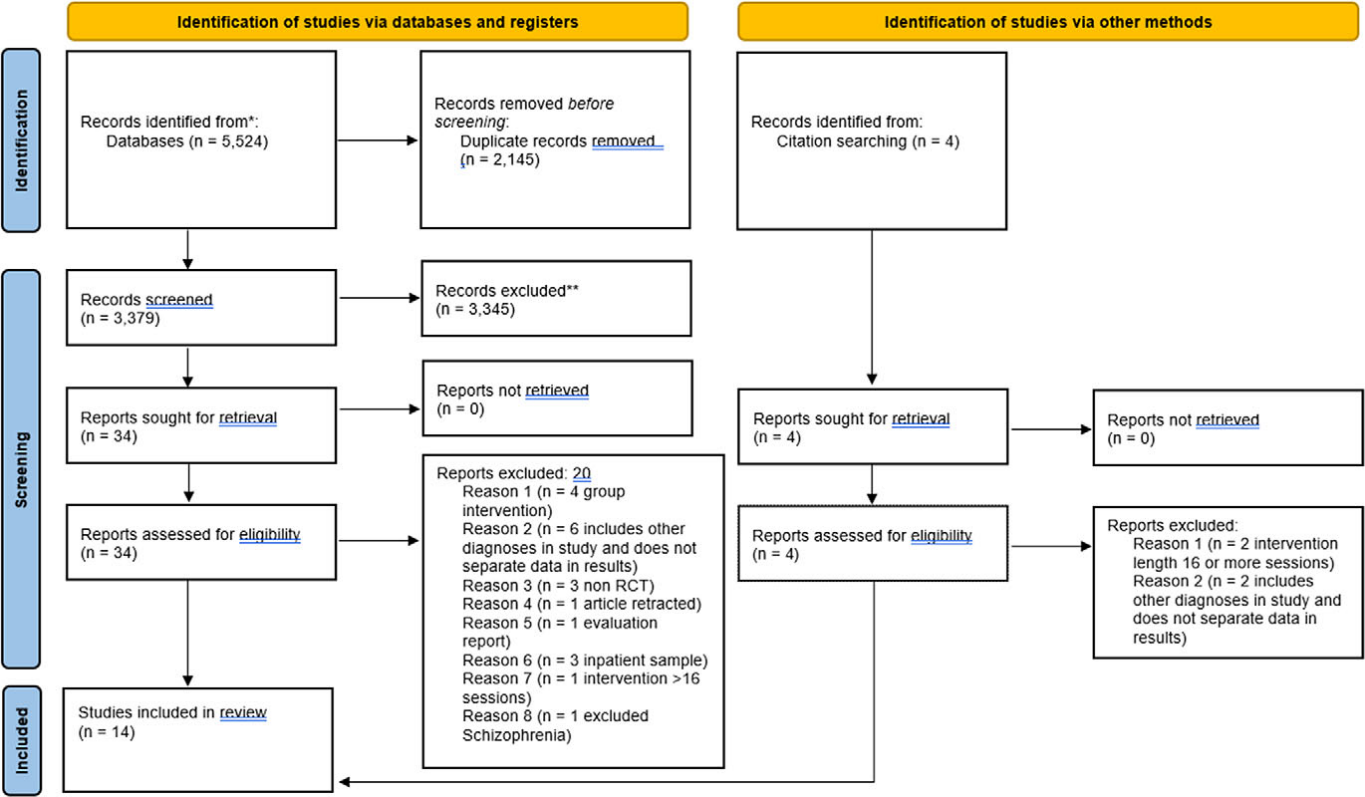
PRISMA flowchart of search results.

PRISMA guidelines informed the search strategy (Page et al., 2021, see Figure 1). Backwards citation searches were completed for included studies, and further potential studies were assessed against the same inclusion and exclusion criteria, which did not result in any further studies being included in the review. An independent reviewer screened 10% of the search results, and any differences were resolved by discussion.

### Data extraction

The main characteristics of each study were extracted and tabulated (see Supplementary Table 1). Three studies did not report effect sizes in their results. For independent samples of equal size, missing effect sizes were calculated using post-intervention means and standard deviation (SD) (Soper, 2024). When post manipulation means or SD were missing, this was requested by contacting the study authors.

### Risk of bias assessment

Each study was assessed using the Revised Cochrane risk-of-bias tool for randomized trials (RoB 2), which includes 28 questions over 5 different domains (Sterne *et al*., 2019). The score range includes: low risk of bias; some concerns – where there are some concerns in at least one domain, but no domain with a high risk of bias; and high risk of bias – where there is a high risk of bias in at least one domain, or the study is judged to have some concerns for multiple domains in a way that substantially lowers confidence in the result.

Thirteen studies scored low risk of bias and one scored some concerns. An independent reviewer repeated the RoB assessment for all fourteen studies. This resulted in agreement in the overall risk of bias rating for each study, which is reported in Supplementary Table 1.

### Statistical analysis

Statistical software MedCalc (https://www.medcalc.org/) was used to conduct all statistical analyses. A random effects meta-analysis was used to integrate effect sizes, due to the heterogeneity of included studies. Statistical heterogeneity was examined and quantified using the *Q* test and *I* ^2^ statistic, with high heterogeneity indicated by a significant *Q* test result (<0.05) or an *I* ^2^ score of over 50%. Publication bias was assessed through visual examination of funnel plots and the results of Egger’s test, with results falling outside of the funnel plot or a significant Egger’s result (<0.05) indicating the presence of publication bias.

## Results

### Summary of studies

Fourteen studies met criteria for inclusion in the review (*n* = 1,382), of which nine were randomized controlled trials (RCT) (*n* = 666), two were pragmatic randomized controlled trials (*n* = 523), one used a Solomon four-group design using randomization (*n* = 80), one was a randomized experimental investigation (*n* = 34), and one was a feasibility RCT (*n* = 79). Twelve studies used a control group of treatment as usual (*n* = 1,223), and two studies used an active control, one of supportive counselling and one with a relaxation intervention (*n* = 159). Six intervention types were used across studies: (1) brief CBT (*k* = 6), (2) memory training (*k* = 2), (3) digital motivation support (*k* = 2), (4) reasoning training module (*k* = 2), (5) psychoeducation (*k* = 1), and (6) virtual reality (*k* = 1). Nine studies used face-to-face interventions, and five used other methods, including 8 weeks of text messages following a single face-to-face session, using an app with an assigned motivation coach for 12 weeks, accessing a single brief computerized reasoning training module, and using virtual reality.

### Summary of effect sizes

In total, 30 clinical outcomes were measured across the 14 studies (see Table 1 for a summary of effect sizes). Large effect sizes were reported for insomnia (*k* = 1); medium to large effect sizes were reported for psychological recovery (CHOICE) (*k* = 3); small to large effect sizes were reported for psychotic symptoms (*k* = 8); medium effect sizes were reported for social comparison (*k* = 1), self-concept (*k* = 1), defeatist beliefs (*k* = 1), self-efficacy (*k* = 1); small to medium effect sizes were reported for depression (*k* = 6), delusions (*k* = 5), paranoia (*k* = 5), quality of life (*k* = 3), motivation (*k* = 2), negative symptoms (*k* = 1); small effect sizes were reported for wellbeing (*k* = 5), anxiety (*k* = 3), functioning (*k* = 2), data gathering (*k* = 2), self-esteem (*k* = 1), subjective goal attainment (*k* = 1), future reward-value representations (*k* = 1), effort-cost computations (*k* = 1), neurocognition (*k* = 1), worry (*k* = 1), rumination (*k* = 1), negative beliefs about self (*k* = 1), suicidal ideation (*k* = 1); and effect sizes were not reported for insight, hospitalization (*k* = 1), social functioning (*k* = 1), self-stigma (*k* = 1). Findings from individual studies, including outcome measures used, are listed in Supplementary Table 1.

**Table 1.** Summary of effect sizes for clinical outcomes

Outcome (number of studies)	Effect size range
Psychotic symptoms (9)	−0.02, 0.91
Depression (6)	0.43, 0.68
Delusions (4)	−0.1, 0.49
Well-being (4)	−0.13, 1.16
Paranoia (4)	0.01, 0.59
Anxiety (3)	−0.21, 0.28
Quality of life (3)	0, 0.5
Psychological recovery (CHOICE) (3)	0.5, 0.87
Functioning (2)	0.09, −0.25
Motivation (2)	−0.14, 0.64
Insight (2)	not reported
Data gathering (2)	0.21, 0.41
Self-esteem (1)	0.16
Subjective goal attainment (1)	1.05
Future reward-value representations (1)	−0.27
Effort-cost computations (1)	−0.11
Negative symptoms (1)	−0.14, −0.66
Neurocognition (1)	−0.1
Hospitalization (1)	Data not reported
Social functioning (1)	Data not reported
Worry (1)	0.47
Rumination (1)	0.32
Self-stigma (1)	Data not reported
Negative beliefs about self (1)	0.24
Social comparison (1)	0.88
Self-concept (1)	0.62
Insomnia (1)	1.9
Defeatist beliefs (1)	0.59
Self-efficacy (1)	0.64
Suicidal ideation (1)	0

## Meta-analysis findings

**Figure 2.**
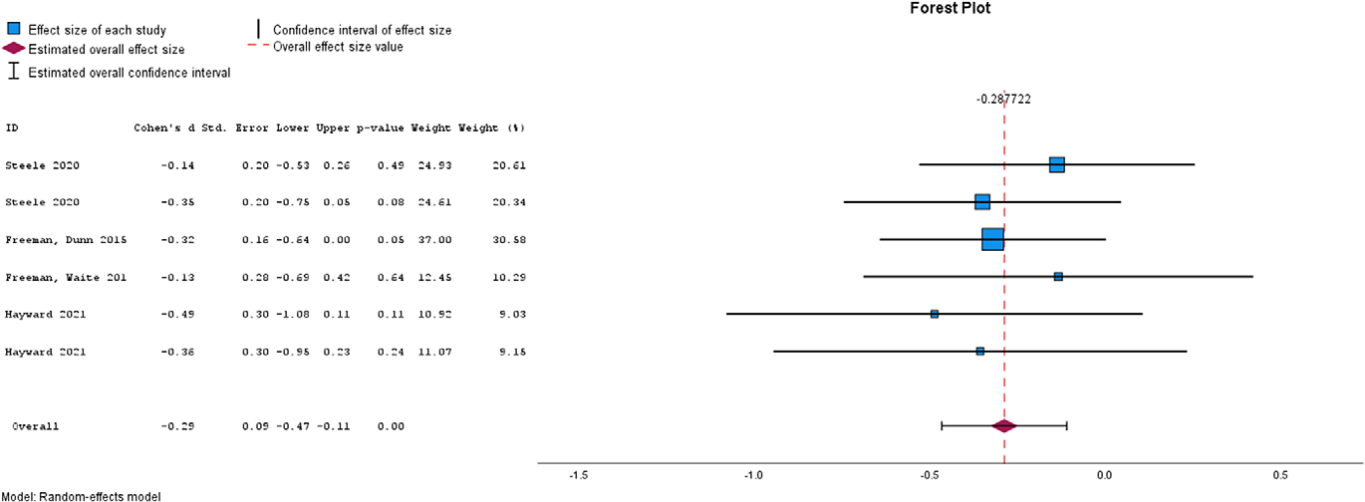
Forest plot for Psychotic Symptoms.

**Figure 3.**
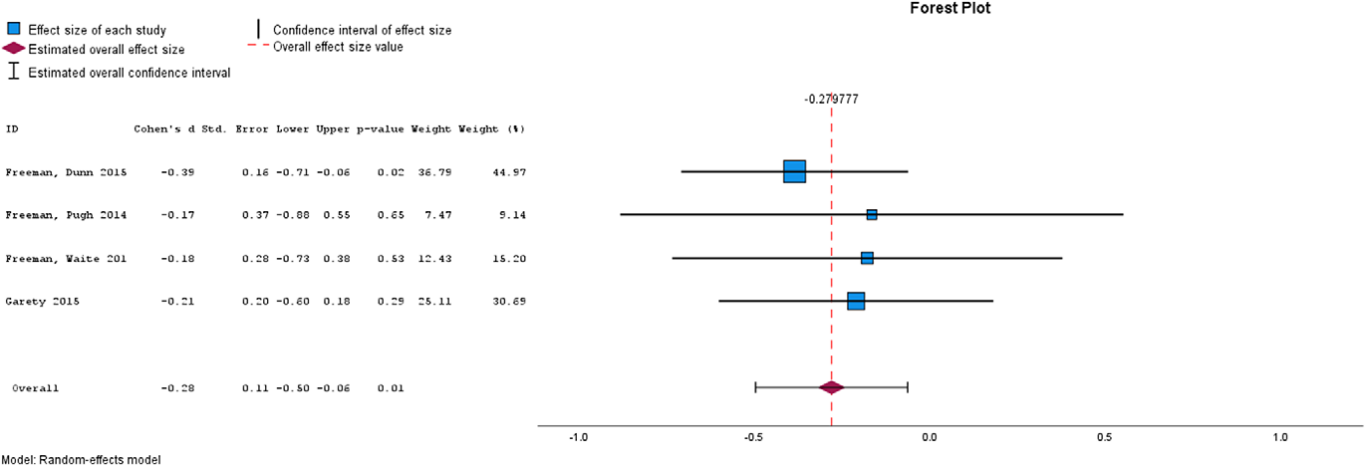
Forest plot for paranoia.

**Figure 4.**
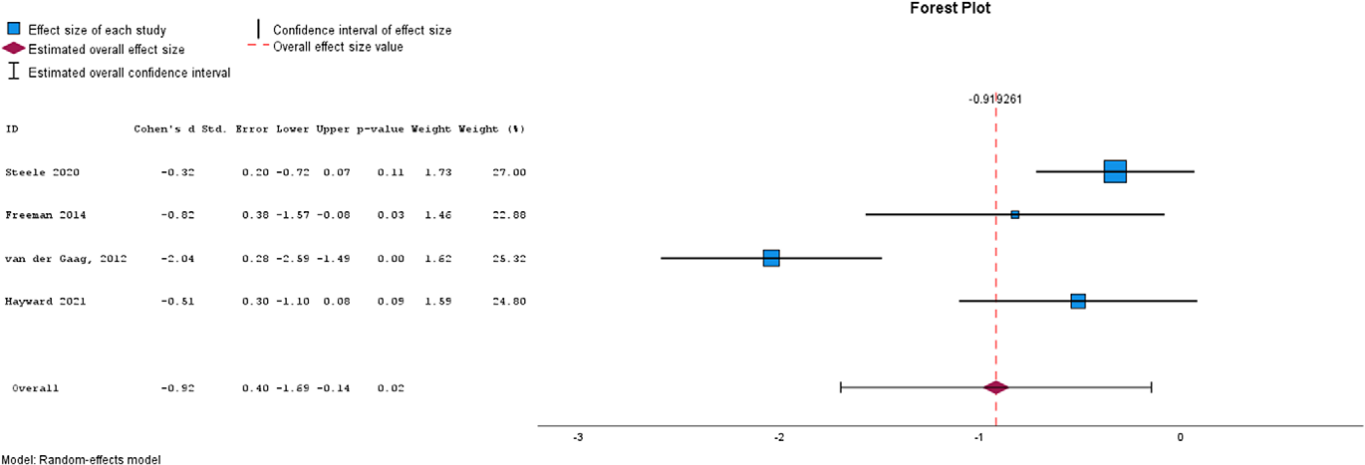
Forest plot for depression.

**Figure 5.**
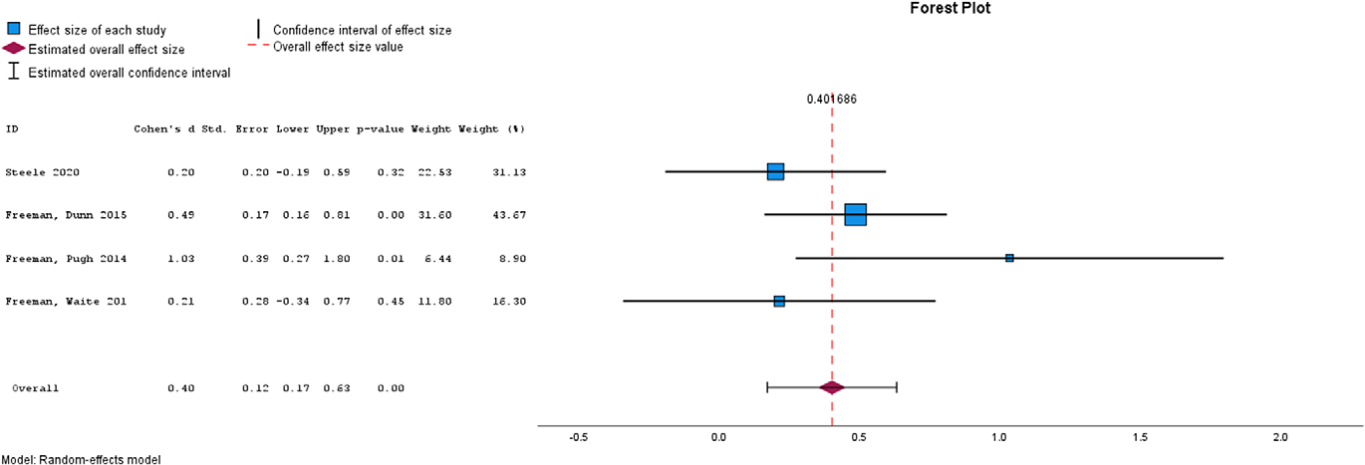
Forest plot for Well-Being.

Meta-analysis was used to assess the effectiveness of brief interventions by clinical outcome (Table 2), and by intervention type (Table 3). Please see Figures 2–5 for the forest plots for each analysis. A separate analysis was conducted for studies using active controls. Meta-analysis was conducted where at least two studies reported the required data (Valentine, Pigott, & Rothstein, 2010). Inspection of funnel plots and the results of Egger’s test identified four analyses showing publication bias, these were for self-esteem, functioning, quality of life, and memory training for depression. Trim and Fill analyses were not undertaken, given only two studies were reported for each of these outcomes and trim and fill is recommended only when at least 10 studies are reported (Mavridis & Salanti, 2014).

**Table 2.** Meta-analysis results by clinical outcome

Clinical outcome	SMD	SE	95% CI	*t*	P	*Q* test	*I* ^2^	Eggers test
Depression	−0.906	0.408	−1.711, −0.102	−2.219	0.027	25.9309**	88.43%	−4.4207
Self-esteem	0.169	0.477	−0.775, 1.114	0.355	0.723	5.2879*	81.09%	5.7396**
Psychotic symptoms	−0.285	0.09	−0.462, −0.108	−3.166	0.002	1.4704	0.00%	−0.2751
Data gathering	0.38	0.121	0.141, 0.619	3.13	0.002	0.8558	0.00%	−0.7373
Anxiety	−0.157	0.202	−0.555, 0.241	−0.777	0.438	3.2245	37.97%	3.5798
Functioning	0.201	0.159	−0.113, 0.516	1.266	0.208	0.3071	0.00%	−2.8454**
Delusions	−0.146	0.132	−0.407, 0.114	−1.106	0.269	4.0008	25.01%	−0.03029
Paranoia	−0.277	0.11	−0.492, −0.0612	−2.526	0.012	0.7779	0.00%	1.2344
Wellbeing	0.405	0.14	0.130, 0.680	2.898	0.004	4.3019	30.26%	1.3716
Quality of life	0.157	0.192	−0.224, 0.538	0.818	0.415	0.7581	0.00%	19.8017**
Active control								
Psychotic symptoms	−0.077	0.153	−0.380, 0.226	−0.503	0.601	0.6072	0.00%	3.5636

*Note:* *p < .05, **p < .001

**Table 3.** Meta analysis results by intervention type

Intervention type and clinical outcome	SMD	SE	95% CI	*t*	P	*Q* test	*I* ^2^	Eggers test
Brief CBT for psychotic symptoms	−0.315	0.117	−0.546, −0.0850	−2.694	0.007	0.7468	0.00%	−0.08767
Reasoning training for data gathering (delusions)	0.38	0.121	0.141, 0.619	3.13	0.002	0.8558	0.00%	−0.7373
Memory training for depression	−1.16	0.848	−2.834, 0.515	−1.367	0.173	24.5469**	95.93%	−21.6467**

*Note:* **p < .0001

### Brief interventions by clinical outcome

Table 2 shows the results from the meta-analyses of the cumulative effect of brief interventions by clinical outcome. Compared with treatment as usual, brief interventions were effective for psychotic symptoms, data gathering, paranoia, well-being, and depression. Brief interventions were not effective for self-esteem, anxiety, delusions, or quality of life. Compared with active control conditions, brief interventions did not appear to be effective for psychotic symptoms, though caution is needed in interpreting this finding given the very small number of studies that have used an active control.

### Brief interventions by intervention type

Table 3 shows the results from the meta-analyses by intervention type. Brief CBT for psychotic symptoms was effective, reasoning training for data gathering was effective, and memory training for depression was not effective.

## Discussion

This study is the first to systematically identify the brief (<16 sessions) psychological interventions that exist for people diagnosed with schizophrenia and to determine their effectiveness using meta-analysis. The review identified fourteen studies, of which 12 compared the brief intervention to treatment as usual and two used an active control. Thirty clinical outcomes were reported on across studies, and six different types of brief intervention were identified, which included brief CBT, memory training, digital motivation support, reasoning training, psychoeducation, and virtual reality. The findings by clinical outcome suggest that brief interventions are effective for psychotic symptoms, paranoia, depression, data-gathering, and well-being. There was no evidence that brief interventions were effective in relation to self-esteem, anxiety, delusions, and quality of life in individuals with schizophrenia. There was no evidence that brief interventions were more effective for psychotic symptoms when compared with active controls, though it is too early to draw any definitive conclusions with only two studies published to date. Meta-analyses by intervention type showed that brief CBT was effective for psychotic symptoms, and reasoning training was effective for data gathering, with no evidence for the effectiveness of memory training for depression. Overall, the evidence in relation to the use of brief interventions for people diagnosed with schizophrenia is encouraging, but caution is warranted in the interpretation of findings from the review given the small number of studies published to date.

The review found small to medium effect sizes for brief CBTp, which supports the findings of Hazell et al. (2016) and is similar to those found for standard CBTp (Jauhar et al., 2014). The findings of this review add to the literature by showing that other types of brief psychological interventions beyond CBT might also be helpful for people diagnosed with schizophrenia. Shorter treatments may be more acceptable for individuals who do not want or who are not ready to engage in longer and/or more intensive therapy (Blenkiron, 1999). Brief interventions also have the potential to result in cost reductions via reduced therapist time, though this would need to be established in future research. Also noteworthy is that two studies included in this review used brief CBT delivered by assistant psychologists (Hayward *et al.*, 2021) and community psychiatric nurses (Turkington, Kingdon & Turner, 2002), and both provided evidence for effectiveness. This suggests that training other professionals to deliver brief CBT to target discreet problems related to schizophrenia could improve accessibility to psychological treatments for this clinical group. The interventions included in this review ranged from a single session to twelve sessions, with two having a single session supported by digital contact (Luther et al., 2020; Schlosser et al., 2018). As with intervention type and target problem, delivery mode and length also need to align with patient preferences, with interventions of shorter duration being selected to suit patient needs (for example cognitive impairment affecting memory and attention), and clinical indication (i.e. a discreet issue where there is an evidence base for the efficacy of the intervention). Furthermore, patient preferences for therapy targets need to be considered, as these may differ from clinician priorities (Loizou, Fowler, & Hayward, 2024).

There are several limitations of the review. Three studies did not provide adequate data to be included in meta-analyses, and whilst study authors were contacted to seek this, data were not provided. Caution is warranted in the interpretation of findings from the review, given the small number of studies included in the meta-analyses (Myung, 2023) and the degree of heterogeneity evident. The studies included in the review were predominantly randomized controlled trials, and it is not clear whether the findings will generalize to routine clinical practice. The effectiveness of other approaches that did not fit the remit for this review, such as Hearing Voices Groups, was not considered. Additionally, the majority of studies used treatment as usual as the control condition, making it difficult to separate the effects of intervention versus nonspecific therapy effects.

The review highlights a number of areas for future research. Although the evidence base for brief interventions is promising, more studies are needed to be able to draw definitive conclusions regarding their effectiveness and potential cost-effectiveness. Future studies also need to determine mediators and moderators of brief interventions to determine which types of interventions work best for different groups, including individuals from diverse and underserved communities. Additionally, qualitative research could explore service users’ experience of brief interventions. The effectiveness of brief interventions available for inpatients with psychosis also warrants attention.

Overall, the evidence to date suggests that brief interventions are effective for reducing psychotic symptoms, paranoia, and depression, and improving well-being in individuals with schizophrenia. In relation to intervention types, brief CBT was effective for psychotic symptoms, and reasoning training was effective for data gathering. Overall, the evidence suggests that brief psychological interventions are effective for several key difficulties associated with schizophrenia, providing an opportunity to improve both access to, and choice of, treatment for individuals with schizophrenia.

## Supporting information

Pike et al. supplementary material Pike et al. supplementary material

## Supplementary material

The supplementary material for this article can be found at http://doi.org/10.1017/S0033291725001126.
