# Supplementary material for: Brief psychological interventions for schizophrenia: a systematic review and meta-analysis
Source: Psychol Med. 2025 May 13;55:e146. doi: 10.1017/S0033291725001126 (PMC12094642; doi:10.1017/S0033291725001126)
Supplement: Pike et al. supplementary material [file S0033291725001126sup001.docx]

**Supplementary Table**

Main characteristics of included studies

| Author and year of publication | Study design | Sample size | Intervention | Comparator | Outcome: primary (p) secondary (s) | Outcome measure | Effect size | P value | Risk of bias |
| --- | --- | --- | --- | --- | --- | --- | --- | --- | --- |
| Steel *et al*  2020 | RCT | 100 | Positive memory training 12 sessions | TAU | Depression (p) | BDI II | 0.43* | 0.03 | Low |
|  |  |  |  |  | Self-esteem (s) | RSES | 0.16 | 0.3 |  |
|  |  |  |  |  | Psychotic symptoms (s) | PANSS | -0.02 | 0.91 |  |
|  |  |  |  |  | Psychotic symptoms (s) | PSYRATS | 0.46 | 0.1 |  |
|  |  |  |  |  | Delusions (s) | PSYRATS | -0.11 | 0.6 |  |
|  |  |  |  |  | Anxiety | GAD7 | 0.26 | 0.11 |  |
|  |  |  |  |  | Functioning (s) | WSAS | -0.25 | 0.22 |  |
|  |  |  |  |  | Wellbeing (s) | WEMWBS | -0.13 | 0.34 |  |
| Luther *et al*  2020 | RCT | 56 | Mobile Enhancement of Motivation in Schizophrenia (MEMS) - one face to face session followed by text contact (three per day) for 8 weeks | Goal setting | Motivation (p) | CAINS (four motivation items) | -0.58* |  | Low |
|  |  |  |  |  | Motivation (p) | QLS motivation index | 0.41 |  |  |
|  |  |  |  |  | Motivation (p) | QLS motivation item | 0.58* |  |  |
|  |  |  |  |  | Motivation (p) | MAP-SR (6 motivation and effort items) | -0.14 |  |  |
|  |  |  |  |  | Goal attainment (p) |  | 1.05*** |  |  |
|  |  |  |  |  | Future reward-value representations (p) | delay-discounting task | -0.27 |  |  |
|  |  |  |  |  | Effort-cost computations (p) | EEfRT | -0.11 |  |  |
|  |  |  |  |  | Negative symptoms (s) | CAINS: Anticipatory pleasure | -0.66* | 0.02 |  |
|  |  |  |  |  | Negative symptoms (s) | CAINS: Past week pleasure | -0.46 | 0.096 |  |
|  |  |  |  |  | Negative symptoms (s) | CAINS: Expressive symptoms | -0.14 | 0.62 |  |
|  |  |  |  |  | Psychotic symptoms (s) | PANSS: Positive symptoms | -0.04 | 0.89 |  |
|  |  |  |  |  | Psychotic symptoms (s) | PANSS: Mood symptoms | -0.02 | 0.94 |  |
|  |  |  |  |  | Neurocognition (s) | BNA | -0.1 | 0.73 |  |
|  |  |  |  |  | Quality of life (s) | WHOQOL | -0.07 | 0.78 |  |
|  |  |  |  |  | Functioning (s) | Strauss-Carpenter Level of Function scale | 0.09 | 0.74 |  |

| Author and year of publication | Study design | Sample size | Intervention | Comparator | Outcome: primary (p) secondary (s) | Outcome measure | Effect size | P value | Risk of bias |
| --- | --- | --- | --- | --- | --- | --- | --- | --- | --- |
| Lui *et al*  2019 | RCT | 80 | Brief CBT, 10 sessions | TAU | Hospitalisation (p) |  |  |  | Low |
|  |  |  |  |  | Relapse (p) |  | * |  |  |
|  |  |  |  |  | Psychotic symptoms (p) | PANSS total | * |  |  |
|  |  |  |  |  | Psychotic symptoms (p) | PANSS positive | * |  |  |
|  |  |  |  |  | Psychotic symptoms (p) | PANSS negative |  |  |  |
|  |  |  |  |  | Psychotic symptoms (p) | PSYRATS | ** |  |  |
|  |  |  |  |  | Insight (s) | SAI |  | 0.203 |  |
|  |  |  |  |  | Social functioning (s) | PSP | * | 0.24 |  |
| Freeman, Dunn *et al*  2015 | RCT | 150 | Worry-reduction CBT intervention over 8 weeks | TAU | Delusions (p) | PSYRATS delusion | 0.49*** | <0.001 | Low |
|  |  |  |  |  | Worry (p) | PSWQ | 0.47*** | <0.001 |  |
|  |  |  |  |  | Delusional distress (s) | PSYRATS distress | 0.41** | 0.001 |  |
|  |  |  |  |  | Psychotic symptoms (s) | PANSS | 0.47*** | <0.001 |  |
|  |  |  |  |  | Paranoia (s) | GPTS | 0.45*** | <0.001 |  |
|  |  |  |  |  | Rumination (s) | PTQ | 0.32* | 0.014 |  |
|  |  |  |  |  | Psychological recovery (CHOICE) (s) | CHOICE | 0.52*** | <0.001 |  |
|  |  |  |  |  | Wellbeing | WEMWBS | 0.23 | 0.03 |  |
| Turkington, Kingdon & Turner  2002 | Pragmatic randomised trial | 422 | CBT - up to 6 sessions delivered by a CPN over 2-3 months | TAU | Overall symptomology (p) | CPRS |  | 0.015 | Low |
|  |  |  |  |  | Insight (p) | Insight rating scale |  | <0.001 |  |
|  |  |  |  |  | Symptoms of Schizophrenia (s) | Schizophrenia change scale |  | 0.003 |  |
|  |  |  |  |  | Depression (s) | MARS |  | 0.258 |  |
| Kumari *et al*  2023 | Solomon four-group design using randomisation | 80 | 30-min individual-based brief psycho-education delivered by a trained nurse | 5-min personalized feedback | Self-stigma (p) | ISMIS | ** | 0.01 | Low |
| Garety *et al*  2015 | Pragmatic randomised trial | 101 | Brief computerized reasoning training intervention | Computer-based activities (inactive with respect to the targeted reasoning processes) of similar duration | Paranoia (p) | State paranoia measure | -0.36* | 0.028 | Low |
|  |  |  |  |  | Improved data gathering and more flexibility in reasoning (s) | JTC–probabilistic reasoning task 60:40 | 0.4* | 0.035 |  |
|  |  |  |  |  | Improved data gathering and more flexibility in reasoning (s) | JTC–probabilistic reasoning task probability of being mistaken | 0.35* | 0.011 |  |

| Author and year of publication | Study design | Sample size | Intervention | Comparator | Outcome: primary (p) secondary (s) | Outcome measure | Effect size | P value | Risk of bias |
| --- | --- | --- | --- | --- | --- | --- | --- | --- | --- |
| Freeman, Pugh *et al*  2014 | RCT | 30 | Brief CBT to reduce negative cognitions about the self. Six sessions over 8 weeks | TAU | Negative beliefs about self (p) | BCSS | 0.24 | 0.353 | Low |
|  |  |  |  |  | Paranoia (p) | GPTS | 0.59 | 0.105 |  |
|  |  |  |  |  | Wellbeing (s) | WEMWBS | 1.16** | 0.007 |  |
|  |  |  |  |  | Social comparison (s) | Social comparison scale | 0.88* | 0.028 |  |
|  |  |  |  |  | Psychotic symptoms (s) | PSYRATS | 0.91 | 0.113 |  |
|  |  |  |  |  | Self-concept (s) | RSQ | 0.62* | 0.017 |  |
|  |  |  |  |  | Anxiety (s) | BAI | -0.21 | 0.507 |  |
|  |  |  |  |  | Depression (s) | BDI II | 0.68 | 0.037 |  |
| Van der Gaag, Van Oosterhout, Daalman, Sommer, & Korrelboom 2012 | RCT | 77 | Competitive memory training (COMET). Delivered in 7 sessions over 2 months | TAU | Depression (p) | BDI II | 0.64** | 0.008 | Low |
|  |  |  |  |  | Auditory hallucinations (s) | PSYRATS - physical characteristics subscale and the negative emotional | 0.3 | 0.197 |  |
|  |  |  |  |  | Auditory hallucinations (s) | PSYRATS - cognitive interpretation subscale | 0.63* | 0.009 |  |
| Freeman, Waite *et al*  2015 | RCT | 50 | CBT for sleep, 8 sessions | TAU | Insomnia (p) | ISI | 1.9 |  | Low |
|  |  |  |  |  | Delusions (p) | PSYRATS | 0.1 |  |  |
|  |  |  |  |  | Hallucinations (p) | PSYRATS | -0.2 |  |  |
|  |  |  |  |  | Sleep quality (s) | PSQI | 0.6 |  |  |
|  |  |  |  |  | Time to sleep onset (s) | Sleep diary | 0.4 |  |  |
|  |  |  |  |  | Total sleep (s) | Sleep diary | 0.2 |  |  |
|  |  |  |  |  | Waking in night (s) | Sleep diary | 0.3 |  |  |
|  |  |  |  |  | Paranoia (s) | GPTS | 0.2 |  |  |
|  |  |  |  |  | Total symptoms (s) | PANSS | 0.2 |  |  |
|  |  |  |  |  | Fatigue (s) | MFI | 0.7 |  |  |
|  |  |  |  |  | Psychological recovery (CHOICE) (s) | CHOICE | 0.5 |  |  |
|  |  |  |  |  | Quality of life (s) | ED-5D-5L | 0.5 |  |  |
|  |  |  |  |  | Wellbeing (s) | WEMWBS | 0.3 |  |  |

| Author and year of publication | Study design | Sample size | Intervention | Comparator | Outcome: primary (p) secondary (s) | Outcome measure | Effect size | P value | Risk of bias |
| --- | --- | --- | --- | --- | --- | --- | --- | --- | --- |
| Schlosser *et al*  2018 | RCT | 43 | PRIME (personalized real-time intervention for motivational enhancement), a mobile-based digital health intervention designed to improve motivation and quality of life. Duration - 12 weeks | Waiting list | Motivation (p) | Trust Task - anticipated pleasure | 0.64* | 0.02 | Some concerns |
|  |  |  |  |  | Motivation (p) | Effort expenditure to increase the likelihood of interactions | 0.58* | 0.3 |  |
|  |  |  |  |  | Defeatist beliefs (s) | MAP-SR | 0.59* | 0.3 |  |
|  |  |  |  |  | Depression (s) | BDI II | 0.63* | 0.3 |  |
|  |  |  |  |  | Self-efficacy (s) | R-SES | 0.64* | 0.2 |  |
| Ross, Freeman, Dunn, & Garety.  2011 | Randomised experimental investigation | 34 | Single 45 min reason training module | Single 45 min attention training module | Data gathering (p) | Beads task 60:40 | 0.41* | 0.012 | Low |
|  |  |  |  |  | Data gathering (p) | Beads task 85:15 | 0.21* | 0.012 |  |
|  |  |  |  |  | Belief flexibility (s) | Maudsley Assessment of Delusions Scale | nr | 0.335 |  |
|  |  |  |  |  | Delusional belief conviction (s) | Percentage rating scale | 0.46 | nr |  |
| Freeman, Lister *et al*  2023 | RCT | 80 | Virtual Reality cognitive therapy, four 30 minute sessions | Virtual Reality mental relaxation, four 30 minute | Persecutory delusion (p) | PSYRATS delusion VAS | 0.18 | 0.69 | Low |
|  |  |  |  |  | Paranoia (s) | GPTS | 0.01 | 0.96 |  |
|  |  |  |  |  | Psychotic symptoms (s) | PSYRATS | 0.2 | 0.43 |  |
|  |  |  |  |  | Avoidance (s) | O-BAT steps avoided | 0.43 | 0.11 |  |
|  |  |  |  |  | Distress (s) | O-BAT distress | 0.05 | 0.93 |  |
|  |  |  |  |  | Number of daily steps (s) | Actigraphy | 0.07 | 0.59 |  |
|  |  |  |  |  | Meaningful activity (s) | Time budget score | 0.13 | 0.56 |  |
|  |  |  |  |  | Quality of life (s) | EQ-5D-5L | 0 | 0.89 |  |
|  |  |  |  |  | Suicidal ideation (s) | C-SSRS | 0 | 0.57 |  |
|  |  |  |  |  | Psychological wellbeing (s) | WEMWBS | 0.01 | 0.87 |  |
|  |  |  |  |  | Recovery (s) | QPR | 0.12 | 0.52 |  |

| Author and year of publication | Study design | Sample size | Intervention | Comparator | Outcome: primary (p) secondary (s) | Outcome measure | Effect size | P value | Risk of bias |
| --- | --- | --- | --- | --- | --- | --- | --- | --- | --- |
| Hayward *et al*  2021 | Feasibility RCT | 79 | Targeted cognitive–behavioural therapy for distressing voices delivered by assistant psychologists, 8 sessions | Supportive counselling, 8 sessions | Psychotic symptoms (p) | HPSVQ- GIVE versus TAU | -0.71 | Nr | Low |
|  |  |  |  | TAU |  | HPSVQ - supportive counselling versus TAU | 0.03 | Nr |  |
|  |  |  |  |  |  | HPSVQ - GIVE versus supportive counselling | -0.75 | Nr |  |
|  |  |  |  |  |  | PSYRATS - GIVE versus TAU | 0.49 | Nr |  |
|  |  |  |  |  |  | PSYRATS - supportive counselling versus TAU | 0.28 | Nr |  |
|  |  |  |  |  |  | PSYRATS - GIVE versus supportive counselling | -0.77 | Nr |  |
|  |  |  |  |  | Depression (s) | HADS - GIVE versus TAU | 0.51 | Nr |  |
|  |  |  |  |  |  | HADS - GIVE versus supportive counselling | 0.73 | Nr |  |
|  |  |  |  |  | Anxiety (s) | HADS - GIVE versus supportive counselling | 0.71 | nr |  |
|  |  |  |  |  |  | HADS - GIVE versus TAU | 0.28 |  |  |
|  |  |  |  |  | Psychological recovery (CHOICE) (s) | CHOICE - GIVE versus TAU | 0.87 |  |  |
|  |  |  |  |  |  | CHOICE - GIVE versus supportive counselling | 0.19 |  |  |

**Significance**: * = P ≤ 0.05, ** = P ≤ 0.01, *** = P ≤ 0.001

**Outcome measures**: BDI II Beck Depression Inventory 2nd edition; RSES Rosenberg Self- Esteem Scale; PANSS Positive and Negative Syndrome Scale; PSYRATS Psychotic Symptom Rating Scale; GAD7 Generalised Anxiety Disorder Assessment; WSAS Work and Social Adjustment Scale; WEMBS Warwick-Edinburgh Mental Wellbeing Scale; CAINS Clinical Assessment Interview for Negative Symptoms; QLS Quality of Life Scale; MAP-SR Motivation and Pleasure Scale; EEfRT Effort Expenditure for Rewards Task; BNA Brief Neurocognitive Assessment; WHOQOL World Health Organization Quality of Life; SAI Schedule for Assessing Insight; PSP Personal and Social Performance Scale; BPRS-24 The Brief Psychiatric Rating Scale expanded version; SLOF Specific Level of Functioning Scale; DPAS The Defeatist Performance Attitude Scale; PSWQ Penn State Worry Questionnaire; GPTS Green et al Paranoid Thoughts Scale; PTQ Perseverative Thinking Questionnaire; CHOICE CHoice of Outcome In Cbt for psychoses; CPRS Comprehensive Psychopathological Rating Scale; MARS Montgomery-Asberg Rating Scale; ISMIS internalized stigma of mental illness scale; JTC Jumping to Conclusions; BCSS Brief Core Schema Scales; RSQ Robson Self-Concept Questionnaire; BAI Beck Anxiety Inventory; ISI Insomnia Severity Index; PSQI Pittsburgh Sleep Quality Index; MFI Multidimensional Fatigue Inventory; EQ–5D Euroqol 5 Dimensions 5 Levels; R-SES Revised Self-Efficacy Scale; MADS Maudsley Assessment of Delusions Scale; VPDS Voice Power Differential Scale; SAPS and SANS Scale for Assessment of Positive and Negative Symptoms; DASS Depression Anxiety and Stress Scale; MANSA Manchester Short Assessment of Quality of Life; O-BAT Oxford-Behavioural Avoidance Task; C-SSRS Columbia Suicide Severity Rating Scale; QPR Questionnaire about the Process of Recovery; HPSVQ Hamilton Program for Schizophrenia Voices Questionnaire
